# Perioperative Outcomes in Patients Who Received Spinal Chloroprocaine for Total Hip or Knee Arthroplasty—Consecutive Case Series Study

**DOI:** 10.3390/jcm11195771

**Published:** 2022-09-29

**Authors:** Khaleifah Alhefeiti, Ana-Maria Patrascu, Sebastien Lustig, Frederic Aubrun, Mikhail Dziadzko

**Affiliations:** 1 Département d’Anesthésie-Réanimation, Hôpital de la Croix-Rousse, Hospices Civils de Lyon, Université Claude Bernard, F-69004 Lyon, France; 2 Département de Chirurgie Orthopédique et Médecine de Sport, Centre d’Excellence FIFA Hôpital de la Croix-Rousse, Hospices Civils de Lyon, Université Claude Bernard, F-69004 Lyon, France; 3 U1290 RESHAPE, INSERM, Université Claude Bernard Lyon 1, F-69003 Lyon, France; 4 Consultation Douleur, Groupement Hospitalier Nord, Hospices Civils de Lyon, F-69004 Lyon, France

**Keywords:** arthroplasty, chloroprocaine, perioperative outcome, spinal anaesthesia

## Abstract

Spinal anaesthesia is an established component of perioperative management for fast-track lower limbs arthroplasty. Short-acting local anaesthetics may present an interesting option for primary non-complicated knee (TKA) and hip (THA) arthroplasty. We describe the perioperative outcomes in patients operated under fixed 50 mg spinal chloroprocaine for total hip and knee replacement. In this retrospective case series study, 65 patients were analysed (median age 65 years, 55% females, benefit from THA (*n* = 31), TKA (*n* = 25), and unicompartmental knee arthroplasty (*n* = 9)). In all cases, anaesthesia duration (87 min) was sufficient for successful surgery (52 min). Up to 45% of patients (THA and less in TKA) developed postoperative pain in the post-anaesthesia care unit (PACU), requiring intravenous morphine titration (up to 7.5 mg). One patient developed severe breakthrough pain requiring advanced regional analgesia. The median PACU stay was up to 97 min (less in TKA), and the incidence of nausea and urinary retention was low. All patients were able to start physical therapy on the same day of surgery. These findings encourage the use of a short-acting agent for spinal anaesthesia in patients with primary non-complicated arthroplasty; however, the relay analgesia should be systematically implemented to avoid breakthrough pain in PACU.

## 1. Introduction

Although the best choice of anaesthesia technique for major orthopaedic surgery is a matter of debate, spinal anaesthesia is an established component of perioperative management for fast-track lower limb arthroplasty [1,2].

Bupivacaine is one of the most common options for spinal anaesthesia in total hip (THA) or total knee (TKA) arthroplasty. It produces a well-known dose-dependent long-acting anaesthesia and analgesia, associated with postoperative urinary retention and delayed motor function recovery, which have led to multiple studies looking for a minimally effective dose, with non-compromising anaesthesia safety and fast-track protocols [3,4]. However, even lower doses of bupivacaine were not constantly associated with a significant improvement in the term of events, precluding the meeting of fast-track protocol requirements [5]. 

The development of modern approaches in hip and knee arthroplasty—such as anterior access in hip surgery, the sub-vastus/medial parapatellar approach in knee surgery, and robot-assisted techniques for both procedures—allowed for a significant reduction in surgical trauma (associated with bleeding, tissue damage, pneumatic tourniquet, and postoperative pain) and procedure time (shortening of surgical time by less than 60 min) [6,7]. A one-night stay or ambulatory setting has become a daily practice in patients with scheduled THA and TKA. Postoperative anaesthesia and analgesia, adapted to the particularities of the patients, surgical trauma, and operating time, must maximize the principles of early rehabilitation and preserve functional capacity.

The arsenal of local anaesthetics used for spinal anaesthesia in orthopaedic surgery includes chloroprocaine, a short-acting local anaesthetic, particularly interesting for scheduled short-duration orthopaedic surgery. The advantages of this molecule include the motor and sensory block of rapid installation, the duration of analgesia allowing surgery of 60 min, and the low rate of adverse effects associated with spinal anaesthesia compared to that with conventional agents (bupivacaine) (hypotension, urinary retention, delay in lifting sensory block).

Spinal anaesthesia with 50 mg of chloroprocaine produces surgical anaesthesia of 60 ± 15 min [8] with respect to outpatient surgery criteria [9]. In our centre, we use spinal chloroprocaine anaesthesia in selected patients scheduled for TKA or THA, and in whom the duration and course of surgery are short and predictable. To date, only one team has recently reported their positive experience of using spinal anaesthesia with chloroprocaine in the context of hip surgery [10].

In this paper, we describe the perioperative outcomes of such patients based on our experience. Our report will be an addition to the literature, to develop anaesthesia techniques adapted to the patient’s journey and integrate surgical particularities.

The main objective of our retrospective study was to analyse the safety and efficacy of short-term spinal anaesthesia (with chloroprocaine) in selected patients who benefit from hip or knee arthroplasty. The secondary objectives were to analyse the characteristics of postoperative analgesia related to the anaesthetic technique, and the events related to this anaesthesia during the postoperative 24 h.

## 2. Materials and Methods

A retrospective study on patients who received spinal anaesthesia for total knee or hip surgery during the years 2020–2021 in a French regional referral centre was performed.

Through the institutional health records system, we identified patients who received spinal anaesthesia with chloroprocaine for elective primary knee or hip arthroplasty. For that, we solicited an Institutional Review Board and sent all identified patients an informed consent form. According to French legislation, if there was no negative response to the use of data for the research, patients were included in the analysed cohort.

We extracted demographic data (age, sex, body mass index, American Society of Anesthesiologists (ASA) status, prostate hypertrophy history), perioperative data (time from spinal anaesthesia to incision, surgical time, blood loss, surgical incidents, the tourniquet use), anaesthesia-related data (level of spinal puncture, sensitive bloc level, time to the motor block, intraoperative need for sedation, bradycardia (heart rate less than 60 per minute), hypotension (mean blood pressure less than 60 or the use of vasopressors)), postoperative data (duration of motor block, the need of supplementary analgesia in post-anaesthesia care unit (PACU), the incidence of moderate to severe pain in PACU, the length of stay (LOS) in PACU, the incidence of postoperative urinary retention and nausea/vomiting in first 24 h), and failure to initiate physical therapy at day 0.

### 2.1. Standard Institutional Protocol for Perianesthesia Management

All patients scheduled for arthroplasty undergo a standard institutional pre-anaesthesia evaluation at least 3 weeks before surgery. Spinal anaesthesia (hyperbaric bupivacaine and sufentanyl), combined with optional regional anaesthesia (such as a single shot or continued adductor canal block for UKA/TKA or ilioinguinal/lateral cutaneous nerve block), and perioperative comfort choice (stepwise choice of sound-isolating earphones, music, distracting virtual reality, or light propofol sedation) is a common approach, used in more than 90% of cases at our institution. A prilocaine–lidocaine patch is systematically used for local analgesia of the lumbar puncture site [11]. Optional anxiolysis, based on Amsterdam Anxiety and Information Scale (APAIS) or a single numeric score, may be administered at the patient’s admission to the hospital or pre-anaesthesia room [12,13]. 

After surgery, patients are admitted to the post-anaesthesia care unit. Additional analgesics are administered as needed. This includes a stepwise administration of acetaminophen, ketoprofen, and morphine titration *pro re nata*. The volume of the bladder is systematically assessed with a bedside ultrasound device (Bladderscan^®^, Verathon Medical BV, Amsterdam, The Netherlands). An evacuator urinary catheter is placed if the bladder volume is more than 500 mL after the failure of spontaneous urination. Discharge criteria are based on the Aldrete score [14], and the Bromage scale [15] is used to assess the resolution of motor block after spinal anaesthesia.

### 2.2. Choice of Patients for Spinal Anesthesia with Chloroprocaine

Patients are systematically informed about the choice of local anaesthetics during the pre-anaesthesia clinics. The ultimate decision to use spinal chloroprocaine is made by an anaesthesiologist after a discussion with the operating surgeon. The following criteria are taken into account: (1) the absence of anticipated surgical difficulties for scheduled primary arthroplasty, (2) compensated comorbidities, and (3) patient compliance. We use a single 50 mg dose of chloroprocaine (Clorotekal^®^, Nordic Group BV, Hoofddorp, The Netherlands) without additives.

### 2.3. Surgical Procedures for TKA, UKA and THA

Total knee arthroplasty is performed using the medial parapatellar approach. THA is performed through the anterior approach. In all cases, a local anaesthetic infiltration (LIA) is used. In TKA/UKA a three times infiltration with Ropivacaine 0.2% is used according to the Kerr and Kohan technique [16]. In THA pericapsular and fascia iliaca infiltration is used with Ropivacaine 0.2%. All arthroplasties were performed by senior surgeons.

### 2.4. Outcomes

The primary outcome was the proportion of patients with successful anaesthesia technique using chloroprocaine. Anaesthesia was deemed to be successful if there was no need to convert spinal anaesthesia to general intraoperatively, to use deep sedation and/or remifentanil for the patient’s discomfort in the surgical site (pain in the surgical site) during the procedure course.

Secondary outcomes were the need for advanced analgesia techniques for the breakthrough pain in PACU (strong opioids and/or regional block, e.g., fascia iliaca compartment or saphenous), and the incidence of PONV and UR in the first 24 h postoperatively.

### 2.5. Statistical Considerations

This is a retrospective non-probability sampling cohort descriptive study where no hypothesis to test was stated. Descriptive quantitative and qualitative statistics are used, data are presented as median [interquartile range, IRQ] or frequency (percentage) as appropriate; non-parametric statistic methods are used for comparisons. A *p*-value less than 0.5 indicates statistical significance. Statistical analyses were performed using JMP 11 (SAS, Cary, NC, USA) software.

### 2.6. Ethics Consideration

Our work received the approval of the institutional ethic committee, CSE-HCL-IRB 00013204, with the reference number 22-729, 25 January 2022, and communicated to the French National Commission on Informatics and Liberty (CNIL), report N 22-5729. All patients included in this study received informed consent for their medical data use. In accordance with French legislation, a written patient’s agreement was not required for any part of the study.

## 3. Results

From 2020 through 2021, sixty-eight patients who received chloroprocaine for THA/TKA/UKA were identified. One month after informed consent was sent, two patients expressed their unwillingness to participate. Medical data for 66 patients were extracted; in one patient spinal anaesthesia failed before the surgery due to a technical problem; finally, 65 patients were included and analysed. Table 1 illustrates patients’ characteristics, principal and supplementary anaesthesia techniques used before the surgery, the onset of anaesthesia, surgical timing, and blood loss.

### 3.1. Primary Outcome

No failed spinal anaesthesia regarding the surgical timing was observed. Sixty-five patients were operated under a single spinal 50 mg chloroprocaine dose without the need for deep sedation or conversion into general anaesthesia.

### 3.2. Secondary Outcomes

In one case the surgeon complained of operative discomfort due to incomplete motor block; however, no conversion to general anaesthesia or deep sedation was required (TKA). Half of the patients received light sedation with propofol (target-controlled infusion, Schnider model for site-effect concentration [17]). Five (4 with THA and 1 with TKA) patients received remifentanil perfusion in the middle of the surgery for painful discomfort beyond the surgical site (spine or shoulder pain due to positioning on the surgical table). Data are reported in Table 2.

Regarding postoperative findings, the time for complete regression of the motor block (charted as Bromage score 0—full flexion of the knee and feet) after 50 mg of chloroprocaine was about 90 min. Up to thirty percent of patients developed pain in the surgical site, requiring morphine titration. One patient (THA) had breakthrough pain requiring postoperative fascia iliaca block. Median PACU LOS was 1 h; however, in patients with THA this time was higher. The incidence of PONV was 4%, and one patient developed urinary retention. All patients were able to start physical therapy on the same day of surgery (Table 3). No transient neurological symptoms related to spinal anaesthesia, LIA, or regional anaesthesia were captured from the electronic health records.

**Figure 1:**
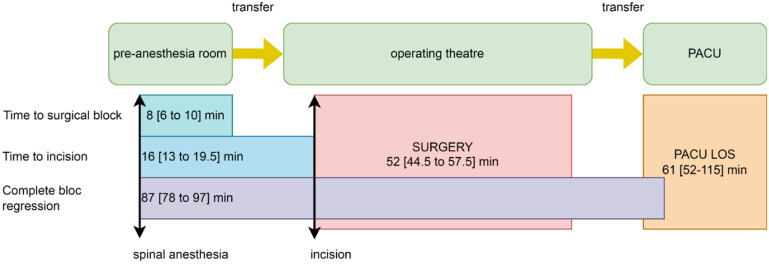
The sequence of anaesthesia, surgery, and PACU in the overall studied population. PACU—post-anaesthesia care unit; LOS—length of stay.

We illustrate the timing of anaesthesia, surgery, and PACU stay in Figure 1.

## 4. Discussion

We report our experience of spinal anaesthesia with a fixed 50 mg dose of chloroprocaine in selected patients, who benefited from non-complicated primary knee or hip arthroplasty with a fast-track protocol. In all patients, the duration of spinal anaesthesia allowed them to accomplish surgery with success and with no need for deep sedation or general anaesthesia. Although plain chloroprocaine for spinal anaesthesia is recommended for procedures not exceeding 40 min [18], several studies have reported its safe use for procedures lasting up to 100 min [19], including hip arthroplasty [10].

Other analysed data are in line with already published outcomes regarding PK/PD-related effects of chloroprocaine (surgical bloc onset, duration, and regression), surgical timing, PACU LOS, PONV, and voiding [9,19,20].

The constant increase in the volume of fast-track and outpatient orthopaedic surgery will predictably lead to the highly controlled optimisation of the anaesthesia care, surgery, and discharge process. For instance, short-acting spinal local anaesthetics were tested and demonstrated a more consistent return of lower-extremity motor function compared to bupivacaine, without a concomitant increase in complications potentially associated with spinal anaesthetics [21,22].

However, we would like to emphasize two points observed in our real-life study.

First, we noticed a significant number of patients who developed pain in the PACU and required morphine titration. The proportion of such patients was two times higher in those who benefited from THA compared to TKA. Moreover, one patient with THA needed an advanced regional analgesia technique for breakthrough pain in PACU. Indeed, chloroprocaine provides a fast onset and relatively short duration, but also the abrupt cessation of a sensitive block because of its high dissociation constant, low partition coefficient, and protein binding and degradation by plasma cholinesterase [23]. As such, a pre-emptive relay analgesia technique should be systematically implemented in patients who benefit from chloroprocaine spinal anaesthesia for short but painful procedures.

All our patients had local infiltration analgesia performed by a surgeon at the end of the procedure. In the case of THA, such a technique probably does not provide 100% relay analgesia. We are not able to conclude if there were some technical flaws while performing LIA; however, the place of systematic preoperative regional analgesia is apparent, with the best technique yet to be defined in both THA and TKA patients.

Another option may be the addition of a spinal short-acting opioid adjuvant to chloroprocaine, such as fentanyl or sufentanyl to extend the time of the analgesic effect [24], but the potential of spinal opioids to produce urinary retention may alter fast-track protocol adherence.

The second point is the changes in the established workflow when chloroprocaine is used for arthroplasty patients (Figure 1). We have a two-bed pre-anaesthesia room adjunct to three orthopaedic operating theatres. In this room, patients are prepared—venous line, standard monitoring, surgical site skin preparation—and receive all types of regional anaesthesia including spinal. Patients are transferred to the operating theatre when the sensitive block is established at least at Th10 level and in-theatre anaesthetic and surgical devices and consumables are ready. 

The quick onset of motor block and limited surgical block duration may put pressure on and increase the workload of paramedical staff. Therefore, managing nurses and clinicians should probably be informed about such particularities, as well as PACU nurses to organize fast discharge.

Regarding the eventual breakthrough pain in PACU, following quick chloroprocaine sensitive block regression, patients should receive additional information about relay analgesia and treatment options available (opioids titration or advanced regional analgesia techniques) in PACU to prevent dissatisfaction. 

Our study has several limitations. It is an observational study with a convenience sample size, with the strength of case series design (high external validity and no interference in the treatment decision process), and well-known limitations (lack of comparison group, susceptible to selection bias). However, the sample size of 60 patients per group yielded 80% power in a randomized trial comparing spinal chloroprocaine and bupivacaine in terms of time-effect [19]. Therefore, our observations may be valid for the duration of the surgical block. For the analysis of secondary outcomes in PACU (PONV, urinary retention, and opioid needs), the number of patients with chloroprocaine is too low to allow a propensity-matched retrospective design, and a prospective randomized controlled study would be methodologically more appropriate. We were not able to analyse the exact causes of breakthrough pain in our patients; however, this study allowed us to improve our local practice and to implement systematic complementary regional analgesia in addition to LIA in all patients operated under chloroprocaine spinal anaesthesia. Transient neurological symptoms associated with anaesthesia techniques were not captured from the electronic health records, and therefore were not evaluated. The workload of clinicians might be interesting to study when comparing the implementation of different anaesthesia techniques. Patients’ opinions and satisfaction were not measured.

## 5. Conclusions

In conclusion, in selected patients undergoing primary knee or hip arthroplasty, the use of spinal anaesthesia with 50 mg of chloroprocaine was successful and safe for the surgery lasting ≤60 min. Up to 45% of patients may experience breakthrough pain in PACU if relay analgesia is not anticipated. These data may be useful for designing controlled studies.

## Figures and Tables

**Table 1:** Demographic, medical, and surgical characteristics (total, breakdown by THA, TKA, UKA).

	Total	THA	TKA	UKA
	*n* = 65	*n* = 31	*n* = 25	*n* = 9
Age	65 [59 to 72]	62.6 [56 to 71]	67 [59.8 to 76]	68 [65 to 71]
Sex, Females	36 (55%)	16 (52%)	17 (68%)	3 (33%)
BMI	24 [21 to 29]	24.3 [20.8 to 28]	23.6 [21.4 to 31]	23.8 [21 to 29]
ASA III	8 (12%)	4 (13%)	3 (12%)	1 (11%)
Hospitalization type (outpatients)	44 (68%)	24 (77%)	14 (56%)	6 (67%)
BPH (males, n = 29)	11 (38%)	2 (13%)	5 (62%)	4 (66%)
LP level	L3-L4 36 (55%)	L3-L4 13 (42%)	L3-L4 17 (68%)	L3-L4 6 (67%)
	L4-L5 29 (45%)	L4-L5 18 (58%)	L4-L5 8 (32%)	L4-L5 3 (33%)
Level of block, Th	10 [8 to 11]	10 [7 to 11]	10 [8 to 11]	10 [8.5 to 11]
Preoperative regional anaesthesia	27 (42%)	3 (10%)	19 (76%)	4(33%)
ACB		n/a	9 (36%)	4 (33%)
C-ACB		n/a	10 (40%)	0 (11%)
LCNB		3 (10%)	n/a	n/a
Time to surgical block	8 [6 to 10]	8 [6 to 10]	7 [6 to 10]	7 [5.5 to 10]
Time to incision	16 [13 to 19.5]	17 [14 to 22]	14 [11 to 18]	14 [11.5 to 16.5]
Length of surgery	52 [44.5 to 57.5]	44 [41 to 48]	55 [52.5 to 62]	58 [51.5 to 66]
Tourniquet use	10 (15.6%)	n/a	1 (4%)	9 (100%)
Blood loss, mL	200 [150 to 300]	200 [105 to 300]	250 [150 to 300]	100 [75 to 225]

THA—total hip arthroplasty; TKA—total knee arthroplasty; UKA—unicompartmental knee arthroplasty. BMI—body mass index; BPH—benign prostatic hyperplasia; LP—lumbar puncture, Th—thoracic level; ACB—adductor canal block; C-ACB—continuous adductor canal block; LCNB—lateral cutaneous nerve block. Data are presented as median [IQR] and frequency (percentage).

**Table 2:** Perioperative secondary outcomes.

	Total	THA	TKA	UKA	*p*
	*n* = 65	*n* = 31	*n* = 25	*n* = 9	
Surgical discomfort	1 (1.5%)	0 (0%)	1 (4%)	0 (0%)	n/a
Hypotension	5 (7.7%)	3 (10%)	2 (8%)	0 (0%)	0.629
No sedation	26 (40%)	11 (35%)	11 (44%)	4 (44%)	0.617
VR	2 (3%)	0 (0%)	1 (4%)	1 (12%)	0.223
Propofol (light sedation)	33 (51%)	17 (55%)	12 (48%)	4 (44%)	0.808
Remifentanil	5 (7%)	4 (13%)	1 (4%)	0 (0%)	0.299

THA—total hip arthroplasty; TKA—total knee arthroplasty; UKA—unicompartmental knee arthroplasty. RA—regional anaesthesia, LP—lumbar puncture; IO—intraoperative, VR—virtual reality glasses. Data are presented as median [IQR] and frequency (percentage).

**Table 3:** Postoperative secondary outcomes.

	Total	THA	TKA	UKA	*p*
	*n* = 65	*n* = 31	*n* = 25	*n* = 9	
Time to complete regression of the motor block, min	87 [78 to 97.5]	84 [76 to 96]	94 [79 to 101]	88 [82 to 93]	0.466
Patients having pain ≥4/10 in PACU	20 (30%)	14 (45%)	5 (20%)	1 (11%)	0.0260
with preoperative RA	5 (8%)	1 (3%)	3 (12%)	1 (11%)	0.759
without preoperative RA	14 (22%)	13 (42%)	1 (4%)	0 (0%)	0.130
Pain level in PACU (out of 10) *	5 [4 to 7]	5 [4 to 7.5]	4 [3.5 to 8.5]	7 [7 to 7]	0.705
Morphine use *, mg	6.5 [5.25–10]	7.5 [4.75 to 7.75]	6 [6 to 10.5]	6 [6 to 6]	0.933
PACU LOS, min	61.4 [52 to 115]	97 [60 to 141]	61.5 [42 to 93]	61 [29.6 to 66]	0.0008
PONV	3 (4%)	1 (3%)	2 (8%)	0 (0%)	0.543
UR	1 (1.5%)	1 (3%)	0 (0%)	0 (0%)	0.573
Fail to initiate PT	0	0	0	0	

THA—total hip arthroplasty; TKA—total knee arthroplasty; UKA—unicompartmental knee arthroplasty. MB—motor block; PACU—post-anaesthesia care unit; RA—regional anaesthesia; PONV—postoperative nausea and vomiting; LOS—length of stay; PONV—postoperative nausea and vomiting; UR—urinary retention. PT—physical therapy. *—data reported for patients with pain >4/10. Data are presented as median [IQR] and frequency (percentage).

## Data Availability

All data generated in this study are the property of the Hospices Civils de Lyon and not will be shared.
